# Knowledge of autism gained by learning from people through a local UK Autism Champion Network: A health and social care professional perspective

**DOI:** 10.1177/13623613231167902

**Published:** 2023-05-02

**Authors:** Louise Kirby, Katy-Louise Payne

**Affiliations:** University of Northampton, UK

**Keywords:** Autism champions, autistic, collaboration, core capabilities framework, learning community, lived experience, training

## Abstract

Few autistic adults perceive that health and social care professionals have good understanding of autism. The countywide Autism Champion Network evaluated here, is an equal partnership of both staff across sectors (Autism Champions) and individuals with lived experience. Autism Champions take knowledge gained back to their teams to support continuous development of services to meet autistic need. This evaluation aims to examine the professional Autism Champions’ perceptions of (1) the knowledge gained through their local network and (2) the enablers and potential challenges of disseminating and applying the acquired knowledge. Seven health and social sector professionals participated in semi-structured interviews with thematic analysis identifying the themes ‘Learning from People’, ‘Makes you think of things in a different light’ and ‘There’s so much going on . . . ’. Results indicated that knowledge of autism gained from networking outside their own team was used and valued more than the professional presentations. It included signposting, sharing resources, using contacts to answer questions, and informal learning from autistic people. These results have implications for developing learning for Tier 2 and above staff. In addition, they could inform the development of future Autism Champion Networks to expand professionals’ knowledge of autism to reduce health and social care inequalities.

**Lay abstract:**

The Autism Act 10 Years On found few autistic adults thought health and social care professionals had a good understanding of autism. Autism training has been made law in the United Kingdom for health and social care staff to tackle health inequality. The county wide Autism Champion Network evaluated here is an equal partnership of interested staff across sectors (Autism Champions) and autistic experts by virtue of lived experience (Autism Advisory Panel). With knowledge flowing both ways, the Autism Champions take learning back to teams to support continuous development of services to meet autistic need. Seven health and social sector professionals from the Network participated in semi-structured interviews on sharing knowledge of autism gained with their teams. All participants provide care and support for autistic people, some working in specialist positions. Results showed that developing new relationships with people outside their own team to signpost to, answer questions and share resources, and informal learning from autistic people, was more valued and used in practice than information gained from presentations. These results have implications in developing learning for those who need above a basic knowledge of autism and may be useful for others considering setting up an Autism Champion Network.

## Introduction

Tackling health inequalities for autistic individuals is a national priority (All Party Parliamentary Group on Autism [APPGA], 2019; NHS England Long Term Plan, 2019) with a training programme on autism for health and care staff made statutory (Health and Care Act, 2022, part 6, 181). Alternative paradigms to the medical model are gaining traction (Burchardt, 2004; López, 2015) advocating difference rather than impairment (Mac Cárthaigh, 2020; O’Dell et al., 2016) and the importance of relationships and quality of life (Osgood, 2020). The onus is on services to reasonably adjust to accommodate autistic individuals where needed (Equality Act, 2010) with a focus on collaboration with autistic people themselves (Brice et al., 2021; Milton, 2014; Shattuck et al., 2020).

The Autism Act 10 Years On (APPGA, 2019) document which informed the National Strategy for autistic children, young people and adults: 2021–2026 (Department of Health and Social Care & Department of Education, 2021) reported that only 32% of autistic adults rated mental health professionals’ understanding of autism as good or very good. Furthermore, only 10% of autistic adults reported that social workers had a good understanding of autism. The Core Capabilities framework for supporting autistic people (Department of Health and Social Care, 2019a, 2019b) describe different tiers of knowledge needed for staff in health, social care and other sectors. Tier 1 is the basic level for everyone, Tier 2 is for those in roles with more responsibility for supporting autistic people and Tier 3 for roles that support more complex situations and may lead services. There is a need to know what knowledge of autism is perceived useful by professionals as autism training is being developed nationally to improve social and health care professionals’ understanding of autism, adapt their practice and make services more accessible to help reduce health inequalities.

Currently autistic people have a shorter life expectancy than neurotypical people (Hirvikoski et al., 2016; Smith DaWalt et al., 2019). Autistic adults experience a significant disparity in health care (Nicolaidis et al., 2015) and are more likely to develop mental ill health but less likely than non-autistic people to get support (Camm-Crosbie et al., 2019). In addition, it appears that autistic people have an elevated risk of victimisation (Weiss and Fardella, 2018) and may be over-represented in the homeless population (Churchard et al., 2019) with 71% of autistic adult survey respondents from across England saying they had unmet social care needs (APPGA, 2019).

The Autism Champion Network was set up in a county in the East Midlands, United Kingdom, to address local reports from autistic individuals that professionals lacked understanding in why they behave as they do. The county is diverse and has a population of 785,200 (Office for National Statistics, 2022). It has large rural areas as well as urban, and areas of deprivation and wealth. Like other counties, numbers of autism diagnoses and those waiting for assessment are increasing illustrating a need for services to further develop to accommodate autistic need throughout their lifetime. From reviewing the literature, no previous research evaluating an Autism Champion Network was found, especially from the perspective of health and social care professionals being the Autism Champions. The Network consists of a non-hierarchical range of professionals across organisations who work with autistic people, alongside people with lived experience of autism, either diagnosed themselves or living with an autistic family member. Any staff member can volunteer to represent their team as an Autism Champion, all that is needed is an interest and acceptance of autism with a desire to increase and share their knowledge. Rather than first attending training then disseminating to teams as Dementia Champions (Mayrhofer et al., 2016) and Cog Champs (Travers et al., 2017), this Autism Champion Network has more in common with parent–professional autism networking groups (Lock et al., 2013). This is because it includes both service users and professionals but in contracts both partners are considered equal with knowledge flowing both ways.

This Autism Champion Network covers all age ranges and is open to representation from all key organisations. Coming together monthly as a community, there are opportunities to learn from presentations and network with others, with an expectation that professionals will take that learning back to their teams. Using an emancipatory approach (Bertilsdotter Rosqvist et al., 2019), freeing the boundaries of a typical community of practice to include those who receive the service, thereby empowering the autistic person to have an equal platform to share their knowledge as autistic, the vision is to improve experiences for autistic people and their families in the county.

Networking is accepted as a method to acquire knowledge (Taba et al., 2017; Wenger, 2010) with human knowledge developed through interactions (Machles et al., 2010) across contexts, collaboration and sharing ideas being key. Practically, however, developing these relationships between groups may not be easy, nor understanding what type of knowledge is useful to be shared (Johnson et al., 2018). The aim of the current research article was to examine the professional Autism Champion’s perception of (1) the knowledge gained through their local network and (2) the enablers and potential challenges of disseminating and applying the acquired knowledge.

Two research questions guided the evaluation:

1. What were the Autism Champions experiences of knowledge exchange within the Autism Champions Network?
2. What are the enablers and barriers to sharing and applying acquired knowledge to improve the experience of autistic service users?

## Method

### Positionality

Reflexivity played an important part throughout the evaluation (Berger, 2015). As the lead and administrator of the network, tacit knowledge and knowing the participants through the network needed active and constant reflection to avoid potential bias. On the contrary, not being part of the teams that the participants were disseminating knowledge to led to a more outsider viewpoint.

To mitigate against bias in the methodology, a colleague with no involvement in the Autism Champion Network but expertise in qualitative methods monitored and reviewed the interview questions and separately analysed a sample of transcripts providing further depth to the analysis.

### Participants

Seven Autism Champions were interviewed representing different health (*n* = 5) and social care services (n = 2). Health sector participants were all from the local NHS Foundation Trust and their occupations are detailed below:

- One nursing clinical assistant from the adult community learning disability team.
- One nurse from the adult community team for learning disabilities.
- Two psychologists from the ADHD Autism Teams (one from adult team; one from children’s team).
- One nurse from the Intensive Support team.

From the social care sector (*n* = 2), the participants occupations are detailed below:

- One adult social services learning disability team assessment and enablement worker.
- One manager of a residential provider for transition age clients.

The occupations of the participants are associated with different levels of pay and education ranging from National Vocational Qualifications (i.e. work-based qualifications) to doctorate level of education. This range in education is representative of the network membership. Table 1 provides the participants ages, time working in the sector and time as an Autism Champion.

**Table 1.** Sample demographic data.

	Mean (*SD*)
Age (years)	43.86 (4.34)
Time working in the sector (years)	17.86 (5.15)
Time as an Autism Champion (months)	16.43 (2.37)

At the time of the evaluation, there were approximately 60 representative Autism Champions across the county, and the intention was that any of these could participate. There were no restrictions relating to age, gender, experience, nationality or specialism. The initial email was sent to the top 16 on the list of Autism Champions ordered from those who had been part of the Network the longest to the least time as an Autism Champion. The intention being that participants would be those with the greatest experience of being an Autism Champion. Six Autism Champions came forward from that invitation but none linking from mental health. A further seventh participant was purposefully recruited from that group to fill this gap, thus the participants were those with more experience of being part of the Network.

Experience of autism for all participants was quite high to extensive (Core Capabilities Framework Tier 2 and Tier 3), all were established in their career and had been an Autism Champion from near the outset of the network in June 2018.

Of these participants six were female and one male. All participants identified as White British. The Autism Champion Network itself is predominantly female and White British but not exclusively so. The literature confirms ethnic minorities being often under-represented in autism initiatives (Hoekstra et al., 2018; Perepa, 2014) and there could be differences in understanding due to cultural norms (Hussein et al., 2019). This is something that warrants further attention but is beyond the scope of this study.

### Ethics

The study was approved by the Faculty of Health, Education and Society Research Ethics Committee at the University of Northampton (approval number FHSRECHEA00212B) and the local NHS Trust.

### Procedure

All participants received an information sheet about the study and had an opportunity to ask questions for 2 weeks prior to participating. Each participant was required to give informed written consent before participation.

All interviews were held face to face in the participant’s own workplace, apart from one who chose to come out of their setting to a familiar Trust meeting room to avoid interruptions. Participants were interviewed once with interviews lasting 20–40 minutes. All interviews were audio recorded with prior permission and transcribed verbatim.

Each interviewee answered similar open questions (see Appendix 1 for interview schedule). The research questions addressed within this paper were (1) What were the Autism Champions experiences of knowledge exchange within the Autism Champions Network? and (2) What are the enablers and barriers to sharing and applying acquired knowledge to improve the experience of autistic service users?

### Data analysis

Analysis was from a non-autistic perspective with open-coded transcripts using thematic analysis (Braun & Clarke, 2006) as an inductive process. Within an interpretive framework, transcripts were read and re-read first coding across all data using NVivo 12 software. Codes were then grouped into themes and sub-themes following an iterative and reflexive stance. Four transcripts were independently coded (CM) to mitigate against bias. The themes were then shared, discussed and agreed.

### Community involvement statement

From the outset, the Autism Champion Network has been a coproduction effort. It was instigated by autistic members of the community stating that they felt they were not understood by professionals. The research results provide a shared opportunity to develop the non-hierarchical community further towards the vision of improving experiences for autistic people and their families.

## Results

The three main themes that emerged from the interviews were (1) learning from people, (2) ‘makes you think of things in a different light’ and (3) ‘there’s so much going on–I guess it just gets lost if you’re not careful’. From all interviews, the benefit of having autistic people as part of the network was highlighted. Consequently, the theme ‘learning from people’ was divided into ‘making links with the others’ and ‘making links with people with autism’

**Figure 1.**
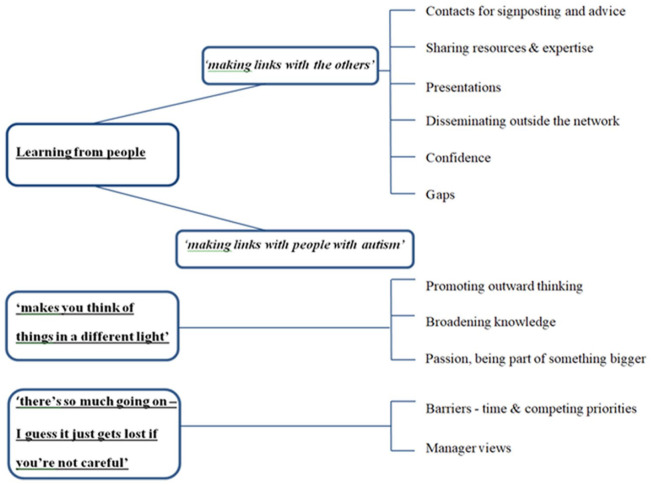
Themes and sub-themes.

As far as possible, the themes use words that participants said, attempting to keep the meanings as close as to what was intended as possible. Although research indicates that autistic people prefer identity-first language (Kenny et al., 2016), professional participants used person-first language, therefore, to remain authentic to what was said this terminology has been used. The themes below are in bold and underlined, sub-themes italicised and in bold (Figure 1).

An extensive overview of the illustrative participant comments for the themes and sub-themes can be found in the supplementary material.

### Learning from people

A strong theme emerged on the advantages of forming links with other professionals from different organisations, ***‘*** making links with the others’. Forming these new relationships enabled professionals to develop a pool of people they could consult with:

It is nice to have a big group of people that I can bounce ideas off. Participant 6

This gives opportunities to obtain feedback on ideas and ask for information either at meetings or using links forged through the network:

now if I’ve got something that I don’t know the answer to I have got a whole group of people that I can ask including quite a few people with um diagnosises themself. Participant 6

As well as developing contacts in other services resulting in knowing where to signpost, sharing resources and expertise, a further positive of linking with the others was described as increased confidence. Participants spoke of consolidating knowledge already held and becoming more comfortable in advocating for people with autism:

I think through networking with so many of you I gained confidence there to stand up to challenge. Participant 5

Thus, making links with the others appeared to have more potential impact on practice for these participants than learning from presentations:

. . .when I was at the meeting last time there was um one of the social prescribers there so I linked up with him and fed that back that to our OTs (occupational therapists) so maybe we can have some kind of joint linking in there. Participant 2

People in the network send links round to each other . . . and er I file it all away ‘cos (laugh) I know it will be useful some day and er yeah pass it on to other people when needed. Participant 6

Although many participants found the professional presentations useful, there was some ambiguity about the pertinence of these presentations and challenges identified to providing professional presentations to such a broad audience.

I think some of the speakers when I’ve been there have been useful, others maybe not so useful. Participant 1

It was suggested that professional presentations were more suitable for those with less knowledge of autism and wondered whether they could be targeted more.

I think probably from my point of view most of the presentations are less relevant because they are more about maybe talking about autism to people who don’t know about autism. Participant 2

if you go to a conference the audience is often very broad and then because you have lots of talks you’re interested in some talks more than others and that’s OK isn’t it. It’s just when there is just one talk. Participant 2

Nevertheless, the breadth of people attending was perceived to be a strength of the Network.

part of the Champions appeal and something that’s really good about it is that it is very broad based. Participant 2

All participants spoke enthusiastically of having autistic individuals as part of the network, ***‘*** making links with people with autism’, this aspect being described as ‘the strongest’, ‘the biggest thing’ and ‘really valuable’. Hearing personal stories seemed powerful and moved knowledge of autism from intellectual to developing real-life understanding:

I think having service users there increases my um awareness of the kinds of day to day issues that impact on them which then helps me to think about that when we’re working with people in our service. Participant 3

One participant described this deeper awareness affecting them emotionally:

I actually felt very very emotional um listening to her story and really learnt a *lot* from what she’d said. Participant 6

It was not just stories that had an impact on the participants’ understanding of autism but how the autistic individual presented information:

seeing him working and seeing how amazingly organised and structured and how he thought and brought that project together, was you know really really amazing. Participant 6

As well as autistic individuals taking equal turns with professionals in speaking every month, they are also equal partners in the network. Professionals and autistic individuals get the opportunity to communicate with each other on a different level away from a service user–practitioner relationship:

it’s *different* from when you are working one to one with them. Participant 6

when somebody is saying it’s this difficult, this is the effect that it has on how I feel about myself and what I can get done, then it brings it alive doesn’t it and that’s at the heart of any of the work we do really. Participant 3

### ‘Makes you think of things in a different light’

Many participants spoke of the knowledge they gained of the wider county and national landscape of autism from attending meetings:

I’ve got a much better knowledge of er what’s going on in the county um and around the country really. Participant 7

This prompted greater outward thinking, for example, in reference to training in autism:

it has really made me think about, instead of going off and doing things on our own, let’s be looking out across the Trust and across other services what we need to be doing. Participant 6

Several participants felt the meetings gave:

a chance to sort of sit back head up and just kind of think more strategically. Participant 3

away from the intense focus on day to day issues in their service.

This could reflect into service issues:

I think it’s made us focus more, so instead of just ticking along and not – having a goal about autism it has made us focus on what we need to do as a team. Participant 6

One participant specifically said that for them the knowledge gained from the network was less clinical but more about ‘who else is out there’ and ‘what else is happening’ (Participant 3).

There was a sense that hearing what they would not normally hear about was a positive for Autism Champions.

This broadening knowledge of autism outside the population served by the professional was not only strategic but also stemmed from the autistic people present themselves:

I hadn’t really thought of people with autism outside of the LD (Learning Disability) population so it has broadened it in that way. Participant 4

A further key aspect of being part of the network seemed to be the feeling of being part of something bigger, all with the same aim and wanting to work together:

. . . you sit back and see everybody’s there because it is more, there’s a passion, they want to make a difference they want to share what they know. Participant 5

With reference to the autistic members:

The service users are there because they really want to be there they are quite vocal, they’ve got different experiences and different reasons why they’re there, you know um and to have that perspective there feels, you know um, very real, not kind of er let’s tick the box of inviting a service user to to this meeting. Participant 3

There was a sense that members felt being part of a group increased chances of success in improving services for autistic users:

like we are saying about a culture change I think we should be a bit more vocal about that because if it going to come from anywhere it will come from the group making noise. Participant 7

### ‘There’s so much going on–I guess it just gets lost if you’re not careful’

Most participants mentioned time and competing priorities as obstacles to fulfilling the representative Autism Champion role. This was also suggested as a reason for gaps in the network from some mental health teams along with not recognising the purpose of the network:

they’ve not responded or just said don’t see the point. Participant 7

. . .until there is a change in the way that people with autism are well until there’s a change of teams that they are put into or until people just kinda suck it up and get on with the job I don’t see a way round. Participant 7

This suggests the need for a culture shift regarding the intersection between autism and mental health. Paediatricians were also a group that was felt missing from the network and there was a

need to get the important people on board. Participant 6

Some participants’ service had experienced a lot of change recently which effected their ability to fulfil the role, but another Autism Champion from a different service had managed through similar change without impacting the role. The difference appeared to be supportiveness of managers. One manager was very supportive, actively encouraged feedback from Autism Champion Network meetings, valuing the Autism Champion role

In our team meetings I am usually asked beforehand if there’s any updates and er my manager and my service manager are both um really up for this um and they are quite happy for me to swan off and do anything I need to for it. Participant 7

This participant is known in their team and beyond and

because I have got this little badge of Autism Champion, I am pretty much constantly getting questions from people in the team asking me about things. Participant 7

From another team experiencing change there appeared not to be active management support

I do think that within our service it is been a priority but I think maybe management don’t always . . . Participant 2

I have spoken about um in supervision that I don’t have time to action the things or to even sometimes read the information or to attend on a regular basis um and about protected time so it was agreed that I could have some protected time but then clinical things always come before protected time . . . Participant 2

Time is clearly a big issue for this Autism Champion. This participant did not think people in their team knew about the Autism Champion role

Since being an Autism Champion no one’s come and asked me about the Autism Champion Network . . . no one’s ever personally approached me and said oh I know you go to have you heard about this or that or what’s your advice on . . . Participant 2

A participant from another team in the same service experienced difficulties in getting people engaged

It’s has been difficult if I’m honest because trying to get people on board. Participant 1

Another explained

because autism is a tiny bit of what we do. Participant 6

Nevertheless, it was acknowledged that

it’s not a lack of want it’s more of a lack of OK how do we do this or organise this. Participant 2

Arising from the independent analysis was a sub-theme around the importance of having a single lead for the network. This was identified as supportive in sustaining engagement.

Other Autism Champions spoke of having feedback from network meetings as a standing item on their team meetings. Another being in a managerial position could influence but needed to explain to their proprietor the value of being part of the network, that

you get outstanding there’s always room for improvement. Participant 5

and

there’s a lot we could still learn including myself so and it’s being involved in things like this that will enable that. Participant 5

This also reflects the passion mentioned earlier that many in the network have. This could be the drive to prevent autism getting ‘lost’, providing motivation to overcome obstacles and leading to proactive dissemination of information, some using personal networks to distribute wider. Participants spoke of their work being ‘more than a job’ and having ‘always been particularly interested in autism’.

## Discussion

The Autism Champion Network was set up to raise professional’s understanding of autism and for them to disseminate this back to teams. Originally it was thought that presentations would be taken back by Autism Champions and delivered to their colleagues. Through seeking to evaluate this, the inductive process of the study identified the type of information professionals found practical to share. While perceptions of learning may not be accurate, themes can be useful in informing improvement (Bond & Lockee, 2018).

**Figure 2.**
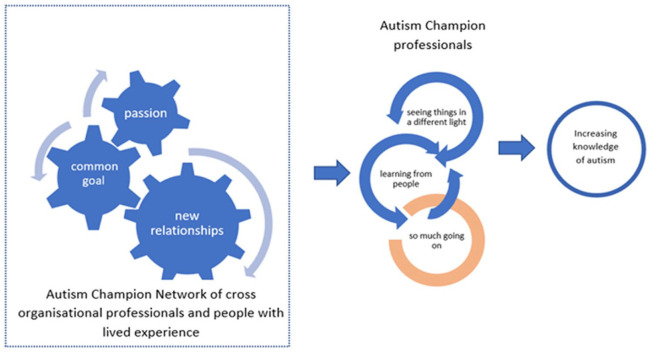
Interactions increase knowledge of autism.

Rather than a teacher–learner relationship from presentations, the learning through the network that participants spoke most enthusiastically of seemed more interconnected, organic and subtle (Figure 2). There is no intended outcome of what knowledge will be gained through this route, but it seems to allow fluidity in learning about autism that accommodates the reality of day-to-day life. However, competing priorities can inhibit learning from people and dissemination of knowledge (Travers et al., 2017).

Research indicates that more than just factual knowledge is valued when considering individuals (e.g. nurses) attitudes to autism (Mac Cárthaigh & López, 2020). The research suggests that looking at autism through a medical lens could result in negative attitudes that disregard abilities. This study demonstrates that professionals interacting with autistic people on a different level through the network (i.e. not viewing the autistic network member as a service user) enables them to see the positives of the condition firsthand which can influence their perception of autism as practitioners.

Learning from contact with people, the results suggest, seemed to promote more behavioural change in the participants than cognitive learning from presentations. Therefore, interactional, relationship-based learning could be beneficial to professionals at this level who want to improve their service for autistic people. Experiencing interactions with a diverse range of professionals and autistic individuals with a common purpose seemed affirmative, appreciating the human element of knowledge development and value-based practice. Professional social network groups supporting acquisition of knowledge is established in the literature (Taba et al., 2017; Wenger, 2010) and ‘autistic expertise’ needed to expand knowledge of autism.

Autistic people do not fit neatly into a homogeneous group (Weiskopf, 2017); therefore, knowledge of the individual and how to interact with the individual is fundamental to clinical practice (Barrett et al., 2015) and needs to be embedded. These results suggest professionals know that learning from sharing experiences and interacting with autistic people is beneficial for practice; therefore, it needs consideration in initiatives to develop knowledge of autism in health and social care staff. The importance of peer learning for developmental disabilities is supported in findings from Duggal et al. (2020) and multidisciplinary network learning for other complex areas (Dalcanale et al., 2011).

Results suggest that professionals were finding that knowledge of autism emerged from their interactions with others, colleagues and autistic people. Learning with people rather than just about them relates to the re-adjustment of power in acquiring human knowledge (Barrett et al., 2015; Bleakley, 2015). Therefore, as well as traditional training, it could be useful for professionals to engage with a network such as this to share expertise and ask questions pertinent to particular situations encountered. Although autism training has been shown to be promising in tackling explicit bias against autism, it has not been shown to reduce implicit bias (Jones et al., 2021). Further research is needed to establish whether interactions within an Autism Champion Network following this model would have influence in reducing any implicit bias against autism.

The results also give insights into what appears important in sustaining the Autism Champion Network. A single lead for the network seemed appreciated. This corresponds to Poocharoen and Ting’s (2015) notion that a visible lead who maintains and manages a network encourages others to join and be involved in the collaborative process. It also seemed clear that manager support was an important driver for success in the Autism Champion role. This indicates that bottom-up passion from individual Champions is not enough alone for them to lead change. Managers who do not value the network nor buy in to the process can form a block to information sharing (Cunningham et al., 2012). Similarly, Mayrhofer et al. (2016) describe manager support as a facilitator for engagement relating to setting up a dementia community of practice.

Crane et al. (2019) report reluctance of mental health services to work with autistic people with a co-occurring mental health condition in their survey of psychiatrists’ knowledge of autism. This is echoed here under the ‘gaps’ theme and could reflect a wider commissioning, organisational gap rather than simply that seen in this network (Crane et al., 2019). Their research suggests that psychiatrists could welcome cross-organisational working and clear signposting. Nevertheless, there may be further barriers to attending network meetings experienced by mental health teams that need further investigation.

## Recommendations for setting up an Autism Champion Network

The results have identified barriers experienced by Autism Champions from this network in engagement and sharing knowledge of autism with their teams. Supportive factors and what Autism Champions value from being part of the network have also been pinpointed. From these, the following pointers are suggested for those wishing to set up a network to broaden professionals’ knowledge of autism:

1. Cross-organisational professionals and autistic individuals all as equal partners.
2. Joint vision that all believe in – to improve experiences for autistic people and their families.
3. Opportunities facilitated for links to be made and different perspectives heard and considered.
4. Managers understand the purpose of the network.
5. Autism Champion to have the support of their manager to take on the role and allowed time to do this.
6. A process for Autism Champions to disseminate knowledge of autism back to their teams.
7. A visible lead to facilitate and administer.

Further sharing of evaluation results could contribute to greater understanding of how Autism Champion Networks can be sustained and succeed in improving experiences for autistic people and their families.

## Limitations

Although the results highlight issues that can be used to develop this network and set up others they need to be interpreted with caution, particularly in generalising to inform training.

The sample might be biased as it was those with a keen interest in autism and the network who took part, all having been involved with the network since its inception. If the study were repeated with participants with less experience of autism, a different emphasis on the importance of types of knowledge of autism gained could result. Furthermore, future research evaluating the Autism Champion Network from the perspective of the autistic individual would be beneficial for both evaluation and future development/recommendations.

The study is rooted in Western sociocultural context, reflecting the Autism Champion Network under evaluation. This needs to be taken into consideration when interpreting the results.

Further research is needed to understand whether networking with other professionals and autistic individuals is of importance at Tiers 2 and 3 to increase knowledge of autism or if it may be less so at Tier 1. Investigation into the impact of e-learning and existing face-to-face learning on developing confidence and understanding in interacting with autistic service users is also required and could provide an interesting comparison.

## Conclusion

This study has highlighted the value professionals who work with autistic people and their families place on developing different kinds of knowledge to improve their practice. In particular, the benefits of learning from people, colleagues and autistic individuals, were emphasised. In order to develop a real understanding of the impact of day-to-day living with autism, interacting with autistic individuals outside the therapeutic relationship was the most powerful. Therefore, developing knowledge of autistic people above developing knowledge of autism is what appeared useful. To accommodate this, engagement in an Autism Champion Network such as this could be considered as an adjunct to traditional training and e-learning on autism for Tier 2 and above.

With autism an NHS priority, these messages are timely for continuing development of training aiming to expand professionals’ knowledge of autism to reduce health and social care inequalities (e.g. access and support) and improve outcomes for autistic people.

## Supplemental Material

sj-docx-1-aut-10.1177_13623613231167902 – Supplemental material for Knowledge of autism gained by learning from people through a local UK Autism Champion Network: A health and social care professional perspective Click here for additional data file. Supplemental material, sj-docx-1-aut-10.1177_13623613231167902 for Knowledge of autism gained by learning from people through a local UK Autism Champion Network: A health and social care professional perspective by Louise Kirby and Katy-Louise Payne in Autism
