# Supplementary material for: Knowledge of autism gained by learning from people through a local UK Autism Champion Network: A health and social care professional perspective
Source: Autism. 2023 May 2;27(8):2507–17. doi: 10.1177/13623613231167902 (PMC10576896; doi:10.1177/13623613231167902)
Supplement: sj-docx-1-aut-10.1177_13623613231167902 – Supplemental material for Knowledge of autism gained by learning from people through a local UK Autism Champion Network: A health and social care professional perspective [file sj-docx-1-aut-10.1177_13623613231167902.docx]

**Appendix 1.** Interview schedule

Takes approximately an hour to complete. Confirm consent form signed.

You need not answer all the questions, if you do not feel comfortable carrying on with the interview we can stop, no need for an explanation.

Remind: The interview will be audio-recorded, then fully transcribed by the researcher. Kept on password protected computer. You can have a copy of the transcription to check. You can withdraw information up to 14 days from participating.

Confidentiality re-explained with proviso regarding any safeguarding issues needing to be shared.

Any questions? Happy to start?

| **Main questions** | **Secondary questions and probes** |
| --- | --- |
| 1. How long have you been an Autism Champion for your team? |  |
| 1. Which sector do you work in? | Health? Education? Social Care? Charity? … |
| 1. Could you describe your level of knowledge of autism when you joined? | Gained from experience?  Gained from attending training? |
| 1. How would you describe your knowledge of autism now? | Why is this? |
| 1. Since being an Autism Champion how, if at all, have you increased knowledge of autism in your team/ service/ organisation? | Which method do you think supported learning about autism best?  Any impact having Autism Advisory Panel members (autistic individuals and family members of autistic individuals) present at Network meetings?  What outcomes have you seen for staff?  What outcomes have you seen for autistic service users and their families?  Who is involved in decisions on how you increase knowledge of autism? |
| 1. What, if any, have been obstacles in your raising knowledge of autism in your team/ service/ organisation? | If relevant, how, if at all, is this approached in supervision? |
| 1. What, if anything, has supported you to continually raise knowledge of autism in your team/ service/ organisation? | Peers?  Managers?  Time? Resources? |
| 1. Have you found any benefits in being an Autism Champion? | Can you explain this? |
| 1. Is there anything you would like the Autism Champion Network to do differently? | If so, what? |
| 1. What would make the Autism Champion Network more successful? |  |
| 1. Is there anything else you would like to add on this topic? | No prompts at this stage. |

**Appendix 2**. Themes and subthemes from interviews (n=7)

| Theme | Subtheme | Illustrative quotes |
| --- | --- | --- |
| *‘makes you think of things in a different light’*  *LEARNING FROM PEOPLE*  *‘making links with the others’*    *LEARNING FROM PEOPLE*  *‘Making links with people with autism’*  ‘*there’s so much going on - I guess it just gets lost if you’re not careful’* | *Promoting outward thinking*  *Broadening knowledge*  *Passion, being part of something bigger*  *Contacts for signposting and advice*  *Sharing resources and expertise*  *Presentations*  *Disseminating outside network*  *Confidence*  *Gaps*  *barriers –* *time & competing priorities*  *manager views* | “So it has really made me think about, instead of going off and doing things on our own, let’s be looking out across the Trust and across other services what we need to be doing”  “I think just um autism is really high up on the agenda in [Trust] wider services so knowing that and obviously commissioners as well are really um have got their eye on it and what services are out there and what can be improved so I think that really helps, to know that what you are doing is part of a big picture, that we are all trying to work together to improve services and life for people” “I think the benefit is that you get you’re sort of up to date with what is going around in the area because I think a lot of people don’t know.” “I think it is more about an increased knowledge of um sort of broader strategy around autism in the county and what is going on in other um areas really.”  “So I guess I have got a much better knowledge of what is happening in non-health sectors”  “I guess it’s for me it is less about clinical knowledge but more about knowledge of who else is out there, what else is happening” “chance to sort of sit back head up and just kind of think more strategically”  “I think it has really broadened my wider knowledge of autism because I tend to in LD I hadn’t really thought of people with autism outside of the LD population so it has broadened it in that way um in that people that I work with and I think it’s sort of helped with that type of thing.” “it has um improved my wider knowledge like I said earlier of people of autistic people.”  “you sit back and see everybody’s there because it is more, there’s a passion, they want to make a difference they want to share what they know” “The service users are there are there because they really want to be there they are quite vocal, they’ve got different experiences and different reasons why they’re there, you know um and to have that perspective there feels, you know um, very real, not kind of er let’s tick the box of inviting a service user to to this meeting.” “Being part of something, part of something that we’re all got the same aim and all want to work together, is really nice.“ “like we are saying about a culture change I think we should be a bit more vocal about that because if it going to come from anywhere it will come from the group making noise.”  “people just like getting together, catching up with people, swapping numbers, feeding back you know feeling like they are up to date as much as they can be with what’s going on in the different areas that relate to autism.” “at Autism Champions there is people from schools, there is representatives from schools, there is representatives from children’s services and I know on a couple of tables that I’ve sat in in the past and we get discussing” “I also like the network opportunity with different professionals because there are such a range of local people” “I think one of the greatest things is the networking with other people and knowing what’s out there um and then it seals the stamp that it is right to keep advocating for people and keep representing people” “I think because you know whichever sector you work in you get quite engrossed in that sector partly because you are so stretched for time that you just have got your head down doing what you do and you don’t get a chance really to link in with other people and to find who that person is in a completely different service that’s interested in ASD or whatever so the Champions Network for me is a great opportunity to see who those people are and then try and link in with them.” “Depending on who you are sitting at a table with you have a conversation with those people, you link in, you find out a bit more about them, why they are there what they are doing. It also means you can talk a little bit about your team and what you do.” “So it’s just kind of you know you get to know who people are and kind of understand what their drivers are in terms of actually this is a really helpful thing somebody is asking us to do and how can we make things more so it just kind of keeps it on the agenda I guess, which can only be a positive thing for clients”.  “increased networks so knowing that actually if I have got a question around social care that there are specific people who have an interest in autism in that” “got a network through which if someone says de de de de? er I don’t know but I can ask at the next Autism Champions, it is that kind of you know link really” “generally making links with the others and talking to them because now if I’ve got something that I don’t know the answer to I have got a whole group of people that I can ask including quite a few people with um diagnosises themself.” “about the networking so coming back and sharing some of that so when I was at the meeting last time there was um one of the social prescribers there so I linked up with him and fed that back that to our OTs so maybe we can have some kind of joint linking in there.” “It is nice to have a big group of people that I can bounce ideas off “ “it’s tapping into pots of people isn’t it” “if I have got a query I’ll probably most likely to email them and ask for advice, those sorts of networks that build up.” “another talk around they gave some good resources about what, what about me, and it was feelings and emotions…” “I think some of the speakers when I’ve been there have been useful, others maybe not so useful “  “People in the network send links round to eachother … and er I file it all away ‘cos (laugh) I know it will be useful some day and er yeah pass it on to other people when needed” “passing that information on I think because I do pass it on personally um whether it’s a forum whatever.” “for me what the Champions brought and I am going to say at a personal level but I was able to bring out because I am the manager I can take it into the work, I can influence.” “purpose is to take it back to your teams and disseminating” “and people do come to us for help around autism, so that’s good that’s an awareness of who to go to if you need a bit of help or what resources are out there “ “because I have got this little badge of Autism Champion I am pretty much constantly getting questions from people in the team asking me about things” “I’m not sure whether again they don’t really have a clear idea of how we can help … no one’s ever personally approached me and said oh I know you go to have you heard about this or that or what’s your advice on”  “I think through networking with so many of you I gained confidence there to stand up to challenge” “I think maybe what it does more is that people who come feel more confident and comfortable that they have some knowledge around autism so maybe that’s the person that people in their team go to.”  “Need to get important people on board”  “Making links with people with autism as well I think that is really important and obviously provides, gives you something doesn’t it, gives you a like you learn from people… personally.”  “people with autism that’s the strongest I think you know gosh yet what we can do for our clients and it makes you more aware doesn’t it really” “I think it got much more of awareness on how it affects people, listening to personal accounts, I think that is probably the biggest thing.” “I think seeing service users in a different capacity has really helped , that’s been really valuable erm so when they are when you know people are on the table with you when you get talking to them it’s *different* from when you are working one to one with them.” “I do think it’s good with people with lived experience to present as well” “seeing him working and seeing how amazingly organised and structured and how he thought and brought that project together, was you know really really amazing” “I actually felt very very emotional um listening to her story and really learnt a *lot* from what she’d said” “ think it is getting to know people in a different way, for me, it is not a therapeutic relationship, it is a person to person, adult to adult. That is very different from a therapist to a client” “having people there with lived experience who are very happy to share that and talk about the strengths and benefits of the diagnosis but also the challenges and the difficulties um is really important and um my sense is they experience the group as a as a positive network to be part of otherwise I’m guessing people wouldn’t still be coming” “I think having service users there increases my um awareness of the kinds of day to day issues that impact on them which then helps me to think about that when we’re working with people in our service.” “it’s vital that people are there to also give their own experience because I think when somebody is talking about I don’t know it’s difficult to link up with a GP we can kind of you know um think about that from a sort of academic, intellectual perspective but we don’t know what that is actually like and then when somebody is saying it’s this difficult, this is the effect that it has on how I feel about myself and what I can get done, then it brings it alive doesn’t it and that’s at the heart of any of the work we do really” “the autistic people that attended the project and the thoughts and things that they have given.”  *“*the volume of different projects we’ve got” *“* Just seems *so much* away from your caseload that you just need to get on with as well.” “Time (laugh) is the biggest factor and resources, being rushed, um and not having the time to process things if I’m honest.” “because autism is a tiny bit of what we do” “It’s quite hard to keep up with what you’re doing in your own service let alone know what’s going on in the wide network!” “I think that due to time constraints um and caseloads etc etc it’s quite difficult to sort of take that time out” “it’s not a lack of want it’s more of a lack of OK how do we do this or organise this” “It’s has been difficult if I’m honest because trying to get people on board” “In our team meetings I am usually asked beforehand if there’s any updates and er my manager and my service manager are both um really up for this” “my immediate manager, my line manager, is very supportive of me being an Autism Champion absolutely yep encourages in that, encourages that totally. Um and values that and knows that this is really important um for the service and service users.” “a couple of hurdles, challenges myself with my proprietor.” “The team manager is very on board with it. She um so yeah there’s would always be the encouragement to attend different groups like that and to feedback” “I do think that within our service it is been a priority but I think maybe management don’t always…” |
